# Clinical Insights From Legionnaires’ Disease: A Retrospective Analysis of Severe Cases on Account of a Cluster

**DOI:** 10.7759/cureus.91237

**Published:** 2025-08-29

**Authors:** Inês L Freitas, Ana R Branco, Francisco Silva, Énio Pereira, Rogério Corga, Paula Pestana, José Miguel Sá

**Affiliations:** 1 Department of Critical Care Medicine, Unidade Local de Saúde do Alto Minho, Viana do Castelo, PRT

**Keywords:** disease cluster, intensive care unit, intermediate care unit, legionella pneumophila, legionnaires disease, severe legionnaires disease

## Abstract

Background

Legionnaires’ disease is a form of severe pneumonia most commonly caused by the Gram-negative bacterium *Legionella pneumophila*. During a sudden increase in Legionnaires’ disease cases, a regional cluster on the northern coast of Portugal led to multiple cases of severe illness requiring admission to a local intensive medicine department. This epidemiological event provided the opportunity to retrospectively review and analyze all patients with severe Legionnaires’ disease admitted to this department (intensive care units (ICU) and intermediate care units (IMCU)) in order to report the epidemiology, clinical features, treatment strategies, and outcomes of these patients.

Materials and methods

This was a retrospective, observational, single-center study conducted at the ICU and IMCU of a peripheral hospital. All adult patients (aged ≥18 years), admitted to these units during a four-year period from January 1, 2020, to December 31, 2023, with a microbiologically confirmed diagnosis of Legionnaires’ disease, were included. Diagnosis was confirmed by positive urinary antigen test and/or positive polymerase chain reaction analyses.

Results

During the study period, 10 patients with severe Legionnaires’ disease were admitted, most of whom were associated with the 2023 outbreak. Among 17 patients who tested positive at our hospital during that cluster, four (23.5%) required IMCU admission and three (17.6%) ICU admission - a total admission rate of 41.2%. The remaining cases occurred in 2020 (n=1) and 2021 (n=2). The mean age of patients was 63 years (standard deviation (SD): 19.4), and 80% (n=8) were male. Diagnosis was made in all cases by urinary antigen testing and by PCR in two cases (20%). Radiologic lung abnormalities were present in all patients at admission. The average length of stay was 11 days (SD: 5.0) in the ICU and 6.9 days (SD: 6.3) in the IMCU. At least one classical risk factor (e.g., smoking, pulmonary disease, diabetes, or immunosuppression) was present in 60% (n=6). The main cause of admission was respiratory failure (n=9, 90%), but 60% (n=6) of patients also presented multi-organ failure, and 50% (n=5) presented acute kidney injury. No patients developed septic shock. While a smaller subset required invasive mechanical ventilation (n=2, 20%, mean duration 11 days, SD: 4.3), the majority were managed with non-invasive ventilatory strategies or high-flow nasal cannula (n=7, 70%). Regarding antibiotic choice, azithromycin was used in 60% (n=6) of patients. A C-reactive protein decrease by half or more was noted 48 hours after antibiotic treatment initiation in seven (70%) patients. All patients survived to hospital discharge.

Conclusion

This study provides a detailed description of the epidemiology, clinical presentation, management strategies, and outcomes of patients with severe Legionnaires’ disease admitted to a peripheral hospital's intensive medicine department. Although such cases are infrequent, regional outbreaks can place a significant strain on healthcare resources. Our findings highlight that timely diagnosis and appropriate treatment management, even in the presence of severe illness and underlying comorbidities, can lead to favorable clinical outcomes.

## Introduction

Despite advances in diagnosis and treatment, Legionnaires' disease remains a public health challenge due to its potential for rapid progression and association with environmental factors. Portugal has experienced several outbreaks of Legionnaires' disease, with the most significant one occurring in 2014, when 334 cases were microbiologically confirmed [1,2]. Between October and November 2023, another cluster was identified on the northern coast region with several cases requiring hospitalization at the peripheral public hospital of the region. This outbreak served as an opportune moment to retrospectively analyze all severe Legionnaires’ disease cases that were admitted during a four-year time frame in order to report the epidemiology, clinical features, treatment strategies, and outcomes of these patients.

Legionnaires' disease, first identified following an outbreak at an American Legion convention in Philadelphia in 1976, is a severe form of pneumonia caused most commonly by the Gram-negative bacterium *Legionella pneumophila*. Multiple species and serogroups have been identified, with *L. pneumophila* serogroup 1 being the most implicated in human disease [3-5].

These pathogens thrive in freshwater environments and can proliferate in artificial water systems. Infection occurs primarily through inhalation of contaminated aerosols, leading to sporadic cases or outbreaks. Upon inhalation of contaminated aerosolized water droplets, bacteria are phagocytosed by alveolar macrophages. *L. pneumophila* evades host defenses by inhibiting phagosome-lysosome fusion and creates a replicative niche within the phagosome. The ensuing immune response involves pro-inflammatory cytokines, which, although essential for bacterial clearance, can contribute to lung damage and systemic symptoms [3,5,6].

Legionnaires' disease typically presents as a severe pneumonia characterized by fever and respiratory symptoms. Patients may also exhibit nonspecific symptoms, such as myalgia, headache, diarrhea, and confusion. Classical risk factors include smoking, pulmonary disease, diabetes mellitus, or immunosuppression [3,6-8].

Early diagnosis of Legionnaires' disease is essential and is possible through methods such as urinary antigen test, polymerase chain reaction (PCR) analyses, culture, or serology [4,5].

Empirical treatment should begin promptly in suspected cases. Antibiotics targeting intracellular pathogens are preferred, such as macrolides and fluoroquinolones. The duration of therapy typically ranges from five to seven days, with longer courses required in immunocompromised patients or those with complications [3].

The prognosis of Legionnaires' disease depends on factors such as timing of diagnosis, presence of comorbidities, and severity of disease at presentation. The overall mortality rate ranges from 5% to 30% [3,6,9-11].

With this article, we aim to report the epidemiology, clinical features, treatment strategies, and outcomes of patients admitted with Legionnaires’ disease to the intensive medicine department - intensive care unit (ICU) and intermediate (IMCU) care unit - of a peripheral hospital in Portugal.

A poster regarding this work was previously presented at the LIVES European Society of Intensive Care Medicine 2024 on October 10, 2024, and the respective abstract was published in the Intensive Care Medicine Experimental journal in October 2024.

## Materials and methods

Study design

This retrospective observational study was conducted to describe the epidemiology, clinical features, treatment strategies, and outcomes of patients with severe Legionnaires’ disease admitted to the intensive medicine department (comprising both the ICU and IMCU) of a peripheral hospital in northern Portugal.

Study population

We included all adult patients (aged ≥18 years) admitted to the ICU or IMCU during a four-year period from January 1, 2020, to December 31, 2023. In this study, admission to these units defined a severe case. All patients included had a microbiologically confirmed diagnosis of Legionnaires’ disease. Confirmation required a positive urinary antigen test and/or a positive PCR analysis. Patients diagnosed with Legionnaires’ disease outside the defined study period or those who did not require ICU or IMCU admission were excluded from the study.

A consecutive sampling approach was used, including all eligible patients who met the inclusion criteria during the study period.

Data collection and variables

Data were collected retrospectively from the hospital’s electronic medical record system. To ensure accuracy, cases were first identified by searching for Legionnaires’ disease diagnoses within the intensive medicine department database. These were then cross-referenced with the clinical pathology department’s registry of microbiologically confirmed Legionella cases.

Variables regarding demographics, clinical presentation, severity scores (APACHE II, SAPS II, SOFA), laboratory values, treatment provided (antibiotic regimen, use of mechanical ventilation, renal support), and outcomes (length of ICU/IMCU stay, survival) were extracted.

Statistical analysis

Given the small sample size, data were analyzed descriptively, using Statistical Product and Service Solutions (SPSS, version 28.0; IBM SPSS Statistics for Windows, Armonk, NY). Continuous variables were reported as means with standard deviations (SD). Categorical variables were presented as frequencies and percentages. No inferential statistical tests were applied.

Ethical considerations

The present study had access exclusively to pseudo-anonymized data related to routine clinical parameters collected during the normal functioning of the ICU and IMCU. The investigators did not have access to the decryption key and were therefore unable to identify or trace the patients involved. From the outset, the principal investigators only accessed pseudo-anonymized information, thereby safeguarding patient anonymity and ensuring the protection of their clinical data. Due to the retrospective nature of the research and the use of pseudo-anonymized data, there was no requirement to submit the study to the Institutional Ethics Committee.

## Results

**Figure 1:**
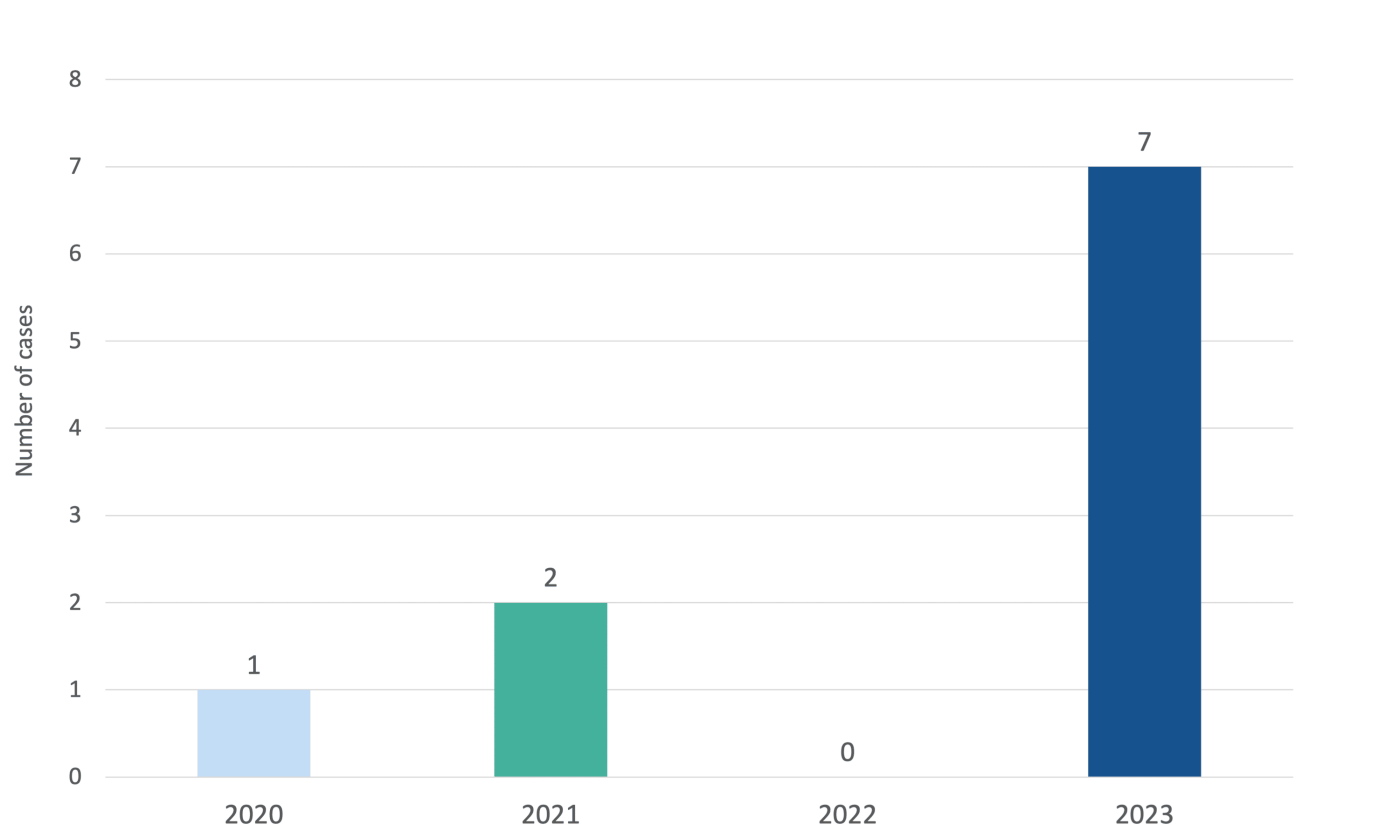
Legionnaires’ disease cases per year.

In total, 10 patients with Legionnaires’ disease were admitted with severe disease to the intensive medicine department in four years (Figure 1). Of the 17 confirmed cases in the 2023 cluster, four (23.5%) were admitted to the IMCU and three (17.6%) to the ICU - with a 41.2% admission rate. The remaining cases occurred in 2020 (n=1, 10%) and 2021 (n=2, 20%).

Regarding the severe Legionnaires’ disease group, patients’ mean age was 63 years (SD: 19.4), and 80% (n=8) were males. Mean APACHE II, SAPS II, and admission SOFA scores were 10.7 (SD: 2.31), 32.2 (SD: 11.58), and 5.1 (SD: 2.42), respectively. Length of stay at the ICU was on average 11 days (SD: 5.0) and at the IMCU 6.9 days (SD: 6.26) (Table 1).

**Table 1:** Characterization of sample patients admitted with severe Legionnaires’ disease over a four-year time period. APACHE - Acute Physiology and Chronic Health Evaluation; ICU - Intensive Care Unit; IMCU - Intermediate Care Unit; SAPS - Simplified Acute Physiology Score; SOFA - Sequential Organ Failure Assessment

Sample Characterization	Results
Total Number of Cases (n)	10
Male (n, %)	8 (80%)
Age (years, mean)	63 (SD: 19.4)
APACHE II (mean)	10.7 (SD: 2.31)
SAPS II (mean)	32.2 (SD: 11.58)
Admission SOFA (mean)	5.1 (SD: 2.42)
ICU Length of Stay (days, mean)	11 (SD: 5.0)
IMCU Length of Stay (days, mean)	6.9 (SD: 6.26)

Diagnosis was made in all cases with a positive urinary antigen test (n=10, 100%) and in two cases with a positive PCR analysis too (n=2, 20%). Radiographic lung changes were present at admission in all patients.

At least one classic risk factor for Legionnaires’ disease was presented by 60% (n=6) of patients - smoking (n=3, 30%), pulmonary disease (n=3, 30%), diabetes mellitus (n=4, 40%), or immunocompromised (n=1, 10%).

At admission to the emergency department, all patients presented respiratory symptoms (n=10, 100%). Four patients (40%) had altered mental status, and nonspecific complaints such as asthenia, anorexia, nausea, or myalgias were reported in 80% (n=8) of cases.

The most common cause of admission to ICU or IMCU was respiratory failure (n=9, 90%), but 60% (n=6) of patients also presented multi-organ failure. Acute kidney injury was found in 50% (n=5) of cases, but renal replacement techniques were not required. No patient evolved to septic shock. Invasive mechanical ventilation was necessary in two cases (20%, mean duration 11 days, SD: 4.3), and non-invasive ventilation or high-flow nasal cannula was used in seven (70%) patients (Table 2).

**Table 2:** Ventilation and oxygenation needs in patients admitted with severe Legionnaires’ disease over a four-year time period.

Ventilation and Oxygenation Need	
Invasive Mechanical Ventilation (n, %)	2 (20%)
Non-invasive Mechanical Ventilation (n, %)	5 (50%)
High-Flow Nasal Cannula (n, %)	6 (60%)
Nasal Cannula (n, %)	9 (90%)

**Figure 2:**
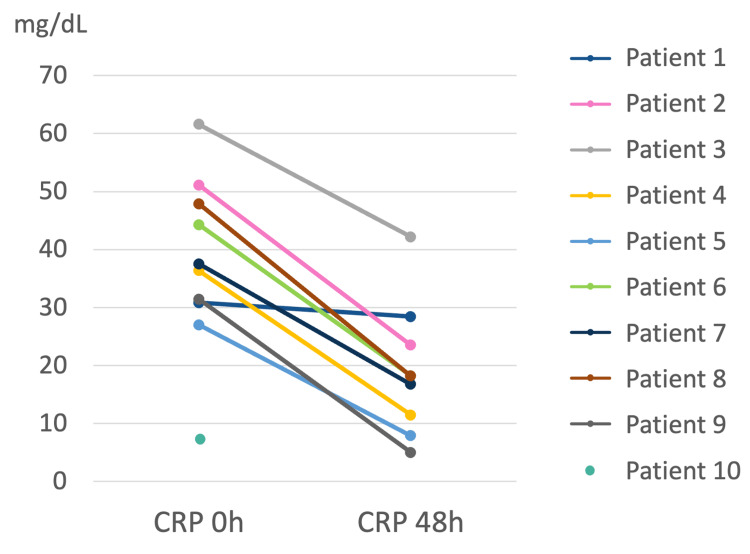
C-reactive protein levels in patients with severe Legionnaires’ disease. C-reactive protein levels by patient at admission (0 h) and 48 hours after directed antibiotic treatment initiation. Note that there was no CRP measurement after 48 hours for patient number 10. CRP - C-Reactive Protein

Regarding antibiotic choice, azithromycin was used in 60% (n=6) of patients and levofloxacin in 40% (n=4). A C-reactive protein (CRP) level decrease by at least half was noted approximately 48 hours after directed antibiotic treatment initiation in seven (70%) patients (Figure 2). The in-hospital survival rate was 100% (n=10).

## Discussion

This study presents a comprehensive retrospective analysis of severe Legionnaires’ disease cases admitted to the ICU and IMCU of a peripheral public hospital in Portugal over a four-year period. The findings provide valuable insights into the epidemiology, clinical presentation, treatment outcomes, and resource utilization associated with this potentially life-threatening condition.

The occurrence of 10 severe cases of Legionnaires’ disease over four years highlights the relatively low incidence of the disease in this region. However, the 2023 cluster, which accounted for 70% of cases, underscores the potential for sudden and unpredictable outbreaks to strain local healthcare resources. The 41.2% admission rate to the intensive medicine department during the outbreak reflects the severity of disease presentation, particularly among vulnerable populations and contrasts with lower admission rates in previous outbreaks [1,2]. Notably, no cases of Legionnaires’ disease were reported in 2022, marking a distinct gap in the otherwise continuous annual case distribution. This fact may be explained by variations in climate and weather conditions, public health measures, or, eventually, a non-detectable form of pathogen by mainstream diagnostic tests that year.

The demographic characteristics of these patients align with well-documented risk profiles for Legionnaires’ disease. The predominance of male patients (80%) and a mean age of 63 years is consistent with prior studies, where advanced age and male sex are recognized risk factors [3,6]. Additionally, 60% of patients presented at least one classic risk factor, such as a history of smoking or pulmonary disease, reaffirming the role of underlying comorbidities in disease susceptibility and severity [3-8].

Respiratory failure was the leading cause of ICU or IMCU admission, affecting 90% of patients. This finding mirrors the established pathophysiology of *L. pneumophila* infections and the observation that all patients exhibited radiological evidence of pneumonia upon admission. Furthermore, multi-organ failure was observed in 60% of patients, highlighting the systemic nature of severe Legionnaires’ disease. The absence of septic shock in this cohort contrasts with some studies reporting higher rates of shock in severe Legionnaires’ disease, suggesting that effective early intervention may have prevented progression to this stage [3,6,10].

Timely initiation of appropriate antibiotic therapy is critical in the management of Legionnaires’ disease. Rapid diagnosis was achieved through urinary antigen testing in all cases, supplemented by PCR in two. In this study, azithromycin and levofloxacin - both first-line agents - were used. A favorable clinical response, indicated by a ≥50% reduction in CRP levels within 48 hours, was observed in 70% of patients.

Respiratory support was tailored according to disease severity. While 70% of patients were successfully managed with non-invasive ventilation or high-flow nasal cannula, only two patients required invasive mechanical ventilation - a much lower result compared to other studies [6]. These findings are encouraging and highlight the importance of close monitoring and tailored respiratory support in managing severe Legionnaires’ disease.

The 100% survival rate observed in this cohort is a particularly noteworthy finding, given that mortality rates for Legionnaires’ disease can range from 5% to 30%, with higher rates reported in ICU settings [3,6,9-11]. Several factors may have contributed to this positive outcome, including early diagnosis facilitated by urinary antigen testing, timely antibiotic administration, and comprehensive supportive care provided in the ICU and IMCU. CRP levels measured were also lower or similar to the threshold associated with increased mortality risk [10]. These results highlight the critical role of early detection and aggressive management in improving patient outcomes.

The 2023 cluster in northern Portugal serves as a stark reminder of the potential burden of Legionnaires’ disease on healthcare systems. While the overall case numbers were modest, the high admission rate to the ICU and IMCU highlights the severity of illness. Local public health’s investigation did not detect an infection source responsible for the cluster.

We believe that this study possesses several notable strengths. First, it provides a comprehensive, real-world analysis of severe Legionnaires’ disease cases over a four-year period, encompassing both endemic and outbreak-related presentations. The study includes detailed data on clinical characteristics, treatment strategies, and outcomes of patients requiring advanced care in ICU and IMCU settings. Second, the use of microbiological confirmation through urinary antigen testing and PCR enhances diagnostic accuracy and case validity. Finally, the 100% survival rate observed - despite the severity of illness - offers valuable insight into the potential effectiveness of early recognition and targeted management in improving patient outcomes.

However, this study presents limitations that should be considered when interpreting the results. First, the small sample size limits the generalizability of the findings. Second, the study was conducted at a single center, which may reflect local epidemiological and clinical practices that are not necessarily representative of other regions or healthcare settings. Third, the retrospective design relies on the accuracy and completeness of existing medical records, which may introduce information bias. Finally, the study provides descriptive insights into clinical features and outcomes and does not allow for comparative statistical analyses. Despite these limitations, the findings offer valuable real-world insights into the management and outcomes of severe Legionnaires’ disease during both endemic and outbreak periods.

## Conclusions

Although Legionnaires’ disease is an uncommon cause of ICU and IMCU admission in our hospital, it remains a significant public health concern, especially during regional outbreaks that can place considerable strain on healthcare resources.

This study provides valuable insights into the clinical and epidemiological characteristics of severe Legionnaires’ disease in a peripheral hospital setting. Our findings underscore the critical importance of early and accurate diagnosis, prompt initiation of targeted antibiotic therapy, and tailored supportive care in improving patient outcomes. Importantly, even in the presence of severe illness and multiple comorbidities, these interventions can lead to favorable clinical results, highlighting the potential to mitigate morbidity and mortality associated with this disease.

## References

[1] Oliveira B, Nora D, Carvalho T, Araujo A, Valente L, Gomes P, Gonçalves-Pereira J (2015). 2014’s Legionnaires’ disease outbreak in Portugal - an intensive care, single-center. 29 patient case series. Intensive Care Med Exp.

[2] Shivaji T, Sousa Pinto C, San-Bento A (2014). A large community outbreak of Legionnaires disease in Vila Franca de Xira, Portugal, October to November 2014. Euro Surveill.

[3] Rello J, Allam C, Ruiz-Spinelli A, Jarraud S (2024). Severe Legionnaires' disease. Ann Intensive Care.

[4] Andrea L, Dicpinigaitis PV, Fazzari MJ, Kapoor S (2021). Legionella pneumonia in the ICU: a tertiary care center experience over 10 years. Crit Care Explor.

[5] Ueda A, Oki M, Yanagi H, Ozawa H, Takagi A (2016). Clinical characteristics of legionella pneumonia diagnosed with Legionella urinary antigen test. Tokai J Exp Clin Med.

[6] Phin N, Parry-Ford F, Harrison T (2014). Epidemiology and clinical management of Legionnaires’ disease. Lancet Infect Dis.

[7] Fields BS, Benson RF, Besser RE (2002). Legionella and Legionnaires' disease: 25 years of investigation. Clin Microbiol Rev.

[8] (2025). Clinical overview of Legionnaires’ disease. Updated.

[9] Che D, Campèse C, Santa-Olalla P, Jacquier G, Bitar D, Bernillon P, Desenclos JC (2008). Sporadic community-acquired Legionnaires' disease in France: a 2-year national matched case-control study. Epidemiol Infect.

[10] Chidiac C, Che D, Pires-Cronenberger S (2012). Factors associated with hospital mortality in community-acquired legionellosis in France. Eur Respir J.

[11] European Centre for Disease Prevention and Control (2025). Legionnaires' disease. Annual Epidemiological Report for 2020.

